# Divergence time estimation using ddRAD data and an isolation-with-migration model applied to water vole populations of *Arvicola*

**DOI:** 10.1038/s41598-022-07877-y

**Published:** 2022-03-08

**Authors:** Alfonso Balmori-de la Puente, Jacint Ventura, Marcos Miñarro, Aitor Somoano, Jody Hey, Jose Castresana

**Affiliations:** 1 Institute of Evolutionary Biology (CSIC-Universitat Pompeu Fabra), Passeig Marítim de la Barceloneta 37, 08003 Barcelona, Spain; 2 Departament de Biologia Animal, de Biologia Vegetal i d’Ecologia, Facultat de Biociències, Universitat Autònoma de Barcelona, 08193 Cerdanyola del Vallès, Barcelona Spain; 3 Àrea de Recerca en Petits Mamífers, Granollers Museum of Natural Sciences, Palaudàries, 102, 08402 Granollers, Barcelona Spain; 4 Servicio Regional de Investigación y Desarrollo Agroalimentario (SERIDA), Ctra AS-267, PK 19, 33300 Villaviciosa, Asturias Spain; 5 Department of Biology, Center for Computational Genetics and Genomics, Temple University, Philadelphia, PA 19122 USA

**Keywords:** Evolution, Evolutionary genetics, Population genetics, Genetics, Next-generation sequencing, Biodiversity

## Abstract

Molecular dating methods of population splits are crucial in evolutionary biology, but they present important difficulties due to the complexity of the genealogical relationships of genes and past migrations between populations. Using the double digest restriction-site associated DNA (ddRAD) technique and an isolation-with-migration (IM) model, we studied the evolutionary history of water vole populations of the genus *Arvicola*, a group of complex evolution with fossorial and semi-aquatic ecotypes. To do this, we first estimated mutation rates of ddRAD loci using a phylogenetic approach. An IM model was then used to estimate split times and other relevant demographic parameters. A set of 300 ddRAD loci that included 85 calibrated loci resulted in good mixing and model convergence. The results showed that the two populations of *A. scherman* present in the Iberian Peninsula split 34 thousand years ago, during the last glaciation. In addition, the much greater divergence from its sister species, *A. amphibius*, may help to clarify the controversial taxonomy of the genus. We conclude that this approach, based on ddRAD data and an IM model, is highly useful for analyzing the origin of populations and species.

## Introduction

Estimating diversification times using genetic data is crucial for analyzing the evolutionary history of species and populations. Divergence time information, in combination with other population parameters, can help not only to understand the origin of biodiversity, but also to delimit species and evolutionary significant units of conservation interest^1^. However, several difficulties arise when trying to obtain accurate split times, specially at shallow divergencies^2^, which has slowed down the adoption of appropriate models for these estimates in populations of non-model species. First, the analysis of divergence occurring at shallow levels is especially problematic due to the effects of coalescence, incomplete lineage sorting, and migration between populations^3,4^, which requires appropriate models for the inferences. Among the most powerful methods for estimating population split times and other demographic parameters are isolation-with-migration (IM) models, which consider coalescence and migration while taking into account mutation rates of the sequence markers used^5–7^. The use of simpler models may lead to important biases in time estimation, particularly of recent splits^8,9^.

Second, the paucity of nucleotide differences between populations makes it necessary to use large sequence datasets in the estimates^10,11^. Reduced-representation genome sequencing methods such as the ddRAD technique^12^ have been widely used in different phylogeographic and fine-scale population structure studies^13,14^. The popularity of this approach is due to the fact that it allows hundreds or thousands of loci to be obtained from a large number of individuals at a moderate cost. The sequence fragments obtained are relatively short (e.g., 145 bp in modern Illumina sequencing systems), and they are generally used only for single nucleotide polymorphism (SNP) discovery, but full ddRAD sequences have enormous potential for using them with IM models. However, they have not so far been combined with these methods and it is not yet clear whether, being so short, they can help produce robust estimates, especially between recently diverged populations and species.

Third, the mutation rates of the markers used should be properly estimated to calculate divergence times. The estimation of rates of novel markers is challenging for several reasons including the variability of mutations rates across different lineages^15,16^ and along the genome^17–19^. Therefore, directly extrapolating mutation rates from distant species or from different genomic regions may lead to deviations in the split-time estimation if they are not properly selected. Since mutation rates of ddRAD markers have not been investigated so far, it is necessary to estimate them, for example, with phylogenetic methods that use molecular clock models^20,21^.

The genus *Arvicola* is a group of Eurasian rodents with a rich evolutionary history and several taxonomic aspects currently under debate^22–25^, which would greatly benefit from the use of an accurate methodology to estimate population split times and other demographic parameters. The target taxa of this work are the montane water vole, *A. scherman* (Shaw, 1801) (formerly *A. terrestris*), and the Eurasian water vole, *A. amphibius* (Linnaeus, 1758). Although these two taxa and their populations have a particularly problematic taxonomy, here we follow the reference of Musser and Carleton^22^, which considers them as two species. These two species form a monophyletic group, while other species of the genus, including two recently described species, are outgroup lineages^26–28^. *Arvicola scherman* is a fossorial species that inhabits lowland and upland grasslands across the main mountainous region of south-western and central Europe^22,29^. Because of their relatively-high population growth and frequent multiannual fluctuations of density, this species is considered a harmful agricultural pest in many areas^30,31^. In the Iberian Peninsula, *A. scherman* has two geographically isolated populations recognized as subspecies, different from the subspecies of central Europe^32^. Specimens from the Cantabrian region (*A. scherman cantabriae*) show differences in skull morphology and have significantly lower body size than that from Pyrenean specimens (*A. scherman monticola*)^32,33^. The close geographic proximity of the two *A. scherman* populations within the Iberian Peninsula makes them an ideal model for studying recent divergence times between populations. Due to the presence of several mountain ranges, it was hypothesized that the Iberian Peninsula was not a single homogeneous refuge during the glaciations and rather that important levels of population structure were generated in different isolated refugia within the peninsula^34^. This refugia-within-refugia hypothesis may be especially true for species of low dispersal capacity such as amphibians, reptiles and small mammals. Obtaining accurate population split times such as those between the Cantabrian and Pyrenean populations of *A. scherman* may be key for testing the refugia-within-refugia hypothesis and understanding whether these populations became isolated due to the effects of glaciations or if their divergence occurred more recently.

*Arvicola amphibius* populates aquatic habitats both in lowlands and mountains from most of Europe (excluding the Iberian Peninsula) to northwestern China^22^. Although most populations are semi-aquatic, there are also fossorial ecotypes^24,25,35^. These two ecotypes are geographically separated but may coexist in some areas of central Europe and cannot be distinguished with mitochondrial data^24^. This species therefore displays a remarkable ecological variability. Paleontological data and analyses of ancestral states and ontogenetic trajectories suggested that the ancestral ecological state of the *Arvicola* genus was aquatic or semi-aquatic while the origin of the fossorial forms was supposed to be relatively recent^36,37^. Due to their ecological versatility and a low mitochondrial divergence between the two lineages, some authors have recently proposed that the populations of *A. scherman* and *A. amphibius* belong to a single species^24^.

In this work, we estimate several important dates and other demographic parameters for the evolutionary history of *Arvicola* using ddRAD data with an IM model, with special emphasis on the analysis of the Iberian populations of *A. scherman*. Thus, we study the phylogeography of the Cantabrian and Pyrenean populations of *A. scherman* and test whether the divergence between them was associated with the glaciations or occurred in more recent times. Additionally, we try to shed some light on the evolution and taxonomic issues of the genus by analyzing the split time and migration rates between the most divergent populations of *A. scherman* as well as between this species and *A. amphibius*. In a first step of the dating procedure, we estimated specific mutation rates for the different ddRAD markers using orthologues from other rodent species. We then employed these rates with an IM model to estimate divergence times, population sizes and migration rates. We show that this framework based on ddRAD data and an IM model allows us to estimate reliable divergence times and other parameters and thus achieve a more in-depth understanding of the generation of phylogeographic patterns and the speciation process.

## Results

### Sequence assembly of ddRAD reads

**Figure 1:**
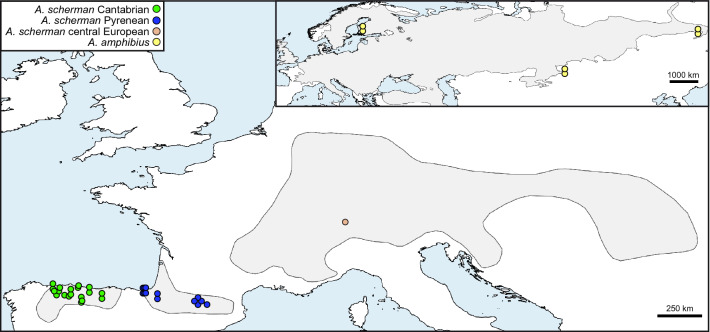
Map showing the samples of *A. scherman* and the European distribution of this species. Note that *A. scherman* is divided into three geographically separated populations: the Cantabrian area, the Pyrenees and central Europe. The map in the upper right shows the samples from the sister species, *A. amphibius*, and its Eurasian distribution. The map was generated using QGIS 2.14^75^ in WGS 84 reference system with distribution areas of both species adapted from the IUCN shapefiles^76,77^ and the land layer downloaded from Natural Earth (http://www.naturalearthdata.com).

A total of 39 specimens of *Arvicola* were analyzed. They included 32 samples from the two Iberian populations of *A. scherman* (19 from the Cantabrian region and 13 from the Pyrenees), 1 sample of the same species from central Europe and 6 samples of *A. amphibius* (Supplementary Table S1 and Fig. 1).

Using the ddRAD protocol^12^, a total of 192,910,996 Illumina reads of 145 bp from the 39 individuals were obtained (Supplementary Table S2). After applying a filter with a tissue samples database to remove any exogenous sequences present in the bone samples used in the study, 71% of the reads remained. Assembly with Stacks^38^ rendered 3361 loci present in all the samples, of which 2877 contained at least one SNP.

The average heterozygosity rate was 1005 and 345 SNPs/Mb for *A. scherman* and *A. amphibius*, respectively.

### Population structure

**Figure 2:**
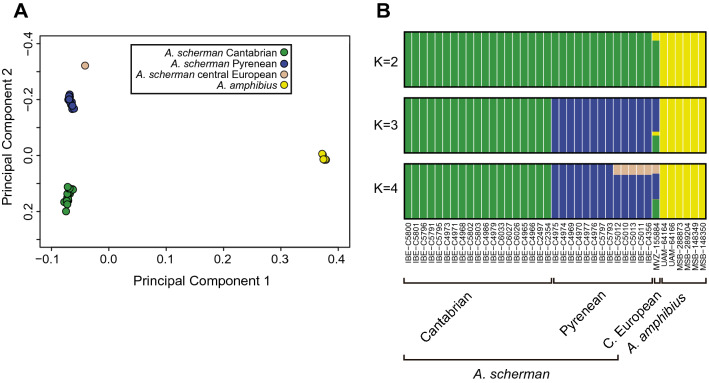
(**A**) PCA of *Arvicola* sp. samples with the 2877 SNPs, in which the first two components explain 27% and 9% of the variance, respectively. (**B**) STRUCTURE analysis plots with the same SNPs and a number of classes (K) from 2 to 4. The samples are ordered in the bar plots by population and geographic longitude.

A principal component analysis (PCA) performed using 2877 SNPs grouped the samples according to both species and the geographical distribution of the populations: it corroborated the genetic separation of *A. scherman* and *A. amphibius* in the first component and of the Cantabrian, Pyrenean and central European populations of *A. scherman* in the second one (Fig. 2A). The STRUCTURE analysis^39^ with two classes (K = 2), which was the best supported model, separated *A. scherman* and *A. amphibius*. The clustering outcome for K = 4 showed a coherent subdivision in the four populations considered here (Fig. 2B). Increasing the K value revealed new components of very low frequency. An F-statistics analysis confirmed pronounced and significant levels of population differentiation between the four populations defined (Supplementary Table S3).

The genome phylogeny based on the average genetic distances between the 3361 loci grouped individuals according to the population of origin (Supplementary Fig. S1), in agreement with the PCA and Structure analyses. In addition, the Cantabrian and Pyrenean populations were closely related, with the central European sample being external to this group. *A. amphibius* appeared as the most external group and highly divergent from *A. scherman*. At the fine-scale level, specimens were grouped according to their locality, indicating the low overall dispersal of the species and the high resolving power of the ddRAD genomic data.

### Estimation of ddRAD loci mutation rates in a phylogenetic framework

In a first step of the dating process, we estimated the specific mutation rates of the ddRAD loci. To do so, we applied a pipeline to find orthologues from each locus in Muroidea, as depicted in Supplementary Fig. S2. Sequences from this superfamily were selected as it includes both the Muridae family, necessary to set the *Mus*–*Rattus* calibration point, and Cricetidae, which contains the *Arvicola* genus. The starting point in the pipeline was the set of variable ddRAD loci present in all the *Arvicola* samples. One sequence per locus was used to perform a BLAST search^40^ against the house mouse (*Mus musculus*) genome. To try to ensure a 1:1 orthology, we considered only sequences with a single hit and an E-value of less than 10^–40^, generating 118 orthologues. Using the sequence coordinates from the mouse sequences detected, 114 mammalian orthologues were downloaded from the ENSEMBL database^41^. Only alignments that included house mouse (*M. musculus*) and brown rat (*Rattus norvegicus*) were kept, leaving a total of 86 orthologues. From these, we selected the available sequences from Muroidea (*Mus pahari*, *Mus caroli*, *Mus spretus*, *Mus musculus*, *Microtus ochrogaster*, *Cricetulus griseus*, *Rattus norvegicus,* and *Peromyscus maniculatus*). We then added the corresponding *A. scherman* sequence to each set of rodent orthologues and realigned them. After filtering out one alignment that was invariant, alignments of 85 loci across 9 rodent species remained (Supplementary Fig. S2).

Using the 85 rodent alignments, we constructed a tree with the BEAST2 program^21^. As a calibration point, we included the mouse-rat split at 10.4–14.0 million years (Myr), based on fossil data^20,42^. The calibrated phylogeny indicated that all the species diverged 18 Myr ago (Supplementary Fig. S3). The mutation rate was obtained for each locus and it was the same for the set of all species as a strict clock was chosen (see “Methods”). The minimum and maximum rates were 0.3 × 10^–9^ and 5.3 × 10^–9^ mutations/site/year, respectively. The average rate of all loci was 2.6 × 10^–9^ and the geometric mean was 2.32 × 10^–9^ mutations/site/year (Supplementary Table S4).

### Application of an isolation-with-migration model to the ddRAD loci

**Figure 3:**
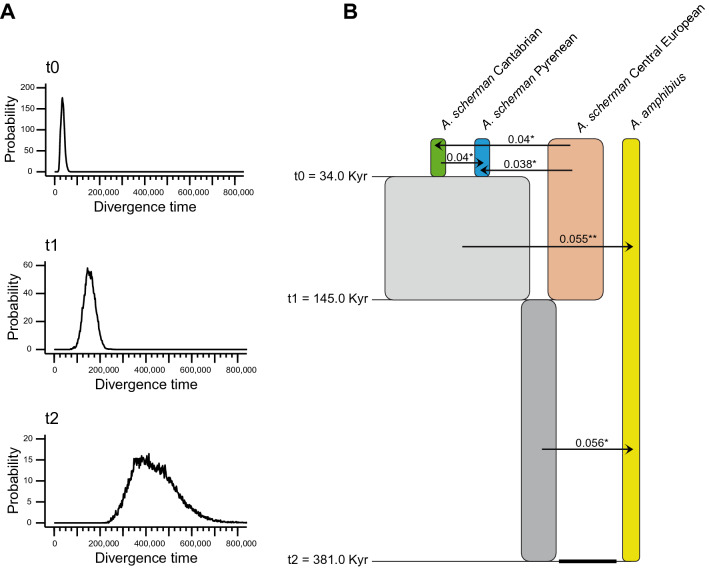
(**A**) Marginal posterior probability histograms of the divergence times obtained from the isolation-with-migration model with four populations. t0 represents the split time between the Cantabrian and Pyrenean populations of *A. scherman*; t1 between the ancestral population of these two and the central European population of the same species; and t2 between the species *A. scherman* and *A. amphibius*. The divergence times and 95% confidence intervals were 34 (17–57), 145 (105–207), and 381 (276–628) Kyr for the three splits, respectively. The divergence in mutation units (scaled for the 145 bp loci) were 0.00975, 0.04125, and 0.1082 for the three splits, respectively. (**B**) Schematic representation of the isolation-with-migration model generated by IMa3. The three split times (t0, t1, and t2) are depicted as solid horizontal lines, with estimated values on the left. Migration arrows indicate statistically significant 2 Nm values (*p < 0.05; **p < 0.01). The width of the boxes is proportional to the estimated population sizes and the ancestral population size is represented by a line.

We applied an IM model implemented in IMa3^43^ to estimate the divergence times between the populations and species of *Arvicola*, as well as the population sizes and migration rates, using the ddRAD loci and the mutation rates of the 85 loci previously estimated (Supplementary Table S4). The mean number of polymorphic positions per locus was 2.29 (Supplementary Fig. S4), a relatively low number, meaning that it was necessary to use a large number of loci for the estimations. IMa3 was tested with different numbers of loci, from less than 100–300, of which 85 were the calibrated loci and the rest were randomly selected from the total pool of variable ddRAD loci. We found that 300 loci were adequate to obtain sufficiently narrow confidence intervals (C.I.) for most parameters while maintaining a good convergence and mixing, as indicated by the similar values for most population size mutation rates and population migration rates obtained from the first and second half of the sampled genealogies (Supplementary Table S5). Probability distributions of divergence times in years (Fig. 3A) as well as of population sizes in demographic units and significant migration rates (Supplementary Fig. S5) were generally continuous and showed a well-defined peak. However, some distributions were noisier and C.I. were relatively wide for some parameters, specifically, the most ancestral time and some of the migration rates. The divergence time between the two Iberian populations of *A. scherman* was 34 thousand years (Kyr) ago, with a 95% C.I. of 17–57 Kyr (Fig. 3A,B); the divergence between the Iberian and central European populations of this species was 145 Kyr ago (C.I.: 105–207); and the divergence between the two species, *A. scherman* and *A. amphibius*, was 381 Kyr ago (C.I.: 276–628). The estimated effective sizes of the Cantabrian, Pyrenean and central European populations of *A. scherman*, and of the *A. amphibius* population, were 51,000, 51,000, 187,000 and 59,000, respectively (Supplementary Table S6). Five significant migration rates involving all present and ancestral populations were found, all with values of an effective number of migrant genes per generation (2 Nm) of ≪ 1 (Fig. 3B).

To test whether the loci with estimated mutation rates were more conserved, as these were selected as having 1:1 orthologues in Muroidea, we used the scalars or relative rates that IMa3 estimates for all loci. The geometric mean of all relative rates was 1.34, being 1.19 and 1.41 for the calibrated and non-calibrated loci, respectively. Thus, the rates were slightly lower for the calibrated loci, as expected, but the distributions mostly overlapped (Supplementary Fig. S6).

## Discussion

### Suitability of ddRAD loci for IMa3 analysis

To study the divergence time between the populations of *Arvicola*, we applied an IM model to the ddRAD data. The length of the markers (145 bp) and the mean number of polymorphic positions (2.29) were small compared to other loci generally used with IM models^44,45^. This resulted in a small number of loci generating parameters with large confidence intervals or not converging properly, as we observed in initial runs. After increasing this number in successive IMa3 runs, we found that a set of 300 ddRAD loci resulted in reasonably good mixing and convergence (Supplementary Table S5), while the distributions were adequate for most of the demographic parameters in the model (Fig. 3A and Supplementary Fig. S5). However, some distributions had relatively wide confidence intervals, probably due to a lack of variability in the loci. Increasing the number of loci slowed the analysis speed, making it impractical. Other ddRAD datasets may require different number of loci and run parameters for achieving adequate convergence and precision, so initial runs are necessary to find the best conditions for each case.

To introduce mutation rates for the divergence-time estimation, we calibrated the ddRAD loci within a phylogenetic framework, obtaining a mean mutation rate for 85 loci of 2.6 × 10^–9^ mutations/site/year. This value is similar to the average rate found for mammals of 2.2–2.6 × 10^–9^ mutations/site/year using the fourfold degenerate sites of genes in different mammalian lineages^46^. However, the germline mutation rates estimated in two different works for the mouse genome using a pedigree approach were 5.4 × 10^–9^^47^ and 6.85 × 10^–9^ mutations/site/generation^48^, respectively. Considering a generation time of 1 year or less, the per-year mutation rate of the whole mouse genome would be more than double or triple that which we determined for the *Arvicola* ddRAD loci. A possibility to explain this discrepancy is that the mouse lineage could be more accelerated than *Arvicola*, although our initial analyses showed that rate variation was low in the rodents' phylogeny (see “Methods”). A more likely explanation for this difference between mutation rates of ddRAD and the whole genome could be in the way in which the ddRAD data is obtained and assembled. On the one hand, we only used and calculated rates from the variable loci, which are 86% of all ddRAD loci in our dataset. On the other hand, it has been shown that ddRAD data do not incorporate the most variable regions of the genome^49,50^ due to the fact that repetitive regions or polymorphic loci for the restriction enzyme sites are not assembled^51,52^. For these reasons, the estimation of specific mutation rates for ddRAD data may be more convenient than extrapolating germline mutation rates from whole genome data. The need to know the generation time to convert per-generation mutation rates to per-year rates is also a major source of uncertainty of the germline mutation rates to estimate divergence times^53^. However, the estimation of mutation rates in a phylogenetic framework may also be affected by other problems such as the availability and suitability of the calibration points^20^ and the delineation of orthology^41,54^. In addition, ddRAD loci for which orthologues were found showed slightly lower relative rates (1.19) compared to the relative rates of all loci (1.34) due to the difficulties in detecting orthologues for the most accelerated loci. Accordingly, the demographic population sizes and split times should be ~ 11% lower than the values estimated here. Moreover, we do not have a reference genome in this group of species, so it was not possible to efficiently filter loci of sex chromosomes or with linkage disequilibrium^55,56^. All these factors in the estimation of mutation rates and their application to the IM model should be taken into account and make it necessary to be cautious with the estimates of split times and other demographic parameters. In this sense, it is convenient that the hypothesis being tested does not refer to a very specific time point but rather to broader time periods.

### Evolutionary history and taxonomy of *Arvicola*

Despite the above-mentioned uncertainties in the estimations, the results obtained with an IM model and the ddRAD data were robust enough to help advance our understanding in some aspects of the evolutionary history of a genus of complex evolution like *Arvicola*. Thus, the ddRAD data allowed us to determine the most likely periods of population and speciation splits from the last 2 million years of Pleistocene glaciations (Fig. 3A,B). The population size was smaller in *A. amphibius* than in *A. scherman* as a whole (Supplementary Table S6), as also reflected in the individual heterozygosity, but both were of the same order of magnitude as those observed for widespread rodents^44^. It is also worth noting the significant migration rates detected between most branches in the tree (Fig. 3B). Although gene flow values were relatively small, some of these migrations could have been important for sharing novel diversity between lineages.

Regarding the two Iberian populations of *A. scherman*, the IM analysis indicated that they diverged 34 Kyr ago (C.I.: 17–57 Kyr) (Fig. 3). These results are consistent with the hypothesis that the split happened during the last glaciation, dated as being between 115 and 20 Kyr ago, in the Late Pleistocene^57,58^. It can then be hypothesized that allopatry could have been initiated within separate refugia during the Last Glacial Maximum or close to this period^58^. Furthermore, the IM model indicated that there was significant gene flow from the Cantabrian to the Pyrenean population, i.e., the two populations exchanged migrants since the initial split, probably as a consequence of range expansion from the original refugia during the Holocene. However, migration rates were small (2 Nm ≪ 1), indicating a low homogenization between the two populations^59^, in line with the strong structure found (Fig. 2) and the morphological differences in both populations, which supports their subspecific status^32,33^. These results are also in agreement with the fundamental role of the refugia-within-refugia hypothesis to explain the generation of biodiversity in the Iberian Peninsula^34^.

As for the other nodes of the population tree, it should be taken into account that we have a much smaller number of samples to properly resolve them. Only one specimen was available for the central European population of *A. scherman* and, for *A. amphibius*, we only had samples from the semi-aquatic populations^24^; we are therefore likely not to have fossorial ecotypes of *A. amphibius*. Hence, the estimated divergence dates and other model parameters as well as the conclusions should be taken with caution. Using the samples available in this work, the IM model allowed us to infer that the Iberian and central European populations of *A. scherman* diverged 145 Kyr ago (C.I.: 105–207). If we assume that *A. scherman* is mainly fossorial, this date marks the minimum age for the origin of this ecotype^37^. It is also interesting that a small amount of gene flow was found from the *A. scherman* ancestral populations (shown in grey in Fig. 3B) to *A. amphibius*, which makes it tempting to speculate on the possibility that these migrations help explain the presence of fossorial ecotypes in *A. amphibius*^25,35^. However, these hypotheses should be tested with a good representation of semi-aquatic and fossorial ecotypes of *A. amphibius* to further analyze the evolution of the ecological forms of *Arvicola*.

The genome-wide data obtained here may also shed some light on the controversy about the species status of *A. scherman* and *A. amphibius*^24,25,28^, assuming that our samples are representative of both. The two taxa appeared clearly separated in the PCA (Fig. 2A), the Structure analysis with K = 2 (Fig. 2B), and the genomic tree (Supplementary Fig. S1). In addition, the IM analysis indicated that both species split 381 Kyr ago (C.I.: 276–628), during the Middle Pleistocene (Fig. 3). We also found evidence of low but significant gene flow between both lineages, although the inclusion of specimens of the two species from overlapping areas may result in a greater amount of gene flow. In view of these results, it seems appropriate to reconsider the recent proposal, based on mitochondrial genetic distances and morphological data, that these populations correspond to a single species^24^. However, further research based on this methodology and with a wider sampling of the known populations of *Arvicola* is advisable to resolve the taxonomic uncertainties.

## Conclusions

In this work, we demonstrate the suitability of a strategy based on ddRAD genomic data together with an advanced IM model, which takes coalescence and migration into account, to estimate the split times of recently diverged populations and closely related species, using the genus *Arvicola* as an example. Ultimately, all the dated nodes of the population tree as well as the estimated population sizes and migration rates provided important insights into different aspects of the evolutionary history and taxonomy of this genus. The ddRAD technique and similar genome reduction approaches are emerging as cost-effective and valid alternatives for generating population genomics data, which can facilitate the future application of this type of sequences along with robust IM dating methods to a wide range of taxa. This is especially important to determine the evolutionary context and the main drivers of population and species divergence in order to better understand the origin of biodiversity. Additionally, with sufficient comparative data, these methods can help to objectively describe different taxa as well as to define evolutionary significant units of conservation importance and, thus, lead to a more precise description of biodiversity.

## Methods

### Samples

Samples of different localities of *A. scherman* and *A. amphibius* were obtained through a combination of our own collections from previous studies^30,31^, loans from museums, and skull bones sampled from barn owl pellets (Supplementary Table S1).

### Ethics statement

No animal was specifically captured for this work. Therefore, this study did not require ethics approval by a specific committee.

### ddRAD library preparation and analysis

DNA extraction from tissues and skull samples was carried out as described in Balmori-de la Puente et al.^60^. To prepare the libraries, we followed the ddRAD protocol^12^ with modifications to process samples independently^14^. Briefly, between 50 and 200 ng of genomic DNA, estimated by qPCR as previously described^14^, was digested with EcoRI and MspI restriction enzymes. Fragments between 300 and 400 bp were selected in a precast EX 2% agarose gel using the E-Gel system (Invitrogen). Different P1 Illumina adapters for each sample (each with a different 5-nucleotide barcode), up to a maximum of 24, and one P2 (the same for all samples) were used. When there were more than 24 samples, different PCR indexes were used. A PCR of 20 cycles was performed with primers annealing over the adapters (or 24 cycles in the case of weak PCR products). Two more PCR reactions were performed to homogenize the coverage of loci. When there was no PCR product, the samples were removed before pooling. The three PCR products from each sample were pooled and visualized in a gel. To construct the final library, the samples were pooled with proportions that depended on the product intensity observed in the gel. After initial bioinformatic analyses to estimate the coverage of each sample, some tissues and most of the skull samples were reprocessed in subsequent libraries to increase coverage. The libraries were sequenced using the NextSeq Sequencing System (Illumina) with the 150-cycles Mid Output kit and single-read runs, at the Genomics Core Facility of the Pompeu Fabra University.

The library sequences were analyzed using different programs in the Stacks 1.35 package^38^. First, *process_radtags* was used to filter the sequences, assign sequences to the different samples according to the sequenced barcode, and truncate them to 145 bp with the recovery option (r). As bone samples of *A. scherman* contained a large proportion of exogeneous sequences, they were filtered using the tissue samples^14^. For this, a database including the sequences of the tissue samples of this species was constructed with the *bowtie-build* tool from Bowtie 2.3.0^61^. We then performed a Bowtie search of the bone sequences against the tissue database with parameters "‐‐score‐min L,‐0.6,‐0.6", retaining only bone sequences that gave at least one hit for further analysis. After this step, sample reads of the same specimen from different libraries were merged by concatenating the sequence files. Using *ustacks* from the Stacks package, the initial minimum coverage (*m*) was set to 3 and the maximum differences between stacks (*M*) to 6. A catalog of loci from all the samples was constructed, allowing for a number of differences between loci from different samples (*n*) of 6 in *cstacks*. After testing different values, this set of parameters was found to be optimal for loci assembly. Finally, the *populations* program from the Stacks package was used with a minimum coverage (*m*) of 6 and a minimum proportion of individuals (*r*) of 1, i.e., only loci that were present in all individuals were selected. The 145 bp sequences were saved in FASTA format (with the command "--fasta_strict") and the first SNP of each variable locus was saved in PLINK format ("--write_single_snp --plink"). The numbers of SNPs and variable loci in FASTA format were not exactly the same (2877 and 2874, respectively), as they were generated with different statistical models. The heterozygosity rate of each individual was calculated by counting the proportion of variable positions along all loci in the FASTA file.

With the aim of detecting any potential bias in the assembly, we performed an Fst analysis for each locus using the SNPs dataset with BayeScan 2.1^62^. The results revealed no major deviations in the assembled loci, with only 4 possible outliers (with slightly higher Fst values) out of the 2877 SNPs.

### Genomic tree and population structure analysis

A genomic tree of the individuals was constructed using all loci, following Igea et al.^8^. To summarize the divergence of the two separate alleles of each locus, a pairwise distance matrix was calculated by estimating genetic distances between all possible combinations of alleles from a pair of individuals using Equation 8.1 in Freedman et al.^63^. Then, the resulting matrix of pairwise distances was used to construct a tree with the Fitch program of the Phylip package^64^. Mid-point rooting was used to represent the tree.

A PCA applied to the SNPs dataset was performed using the program SNPRelate available in R, using the genetic covariance matrix^65^.

STRUCTURE 2.3.4^39^ was applied to the same SNPs. An admixture and uncorrelated allele frequency model with the ancestry prior, recommended when the sampling is unbalanced, was used. The number of populations, K, analyzed ranged from 2 to 6, and the initial Dirichlet parameter for degree of admixture was defined for each K as 1.0 divided by the number of populations. The optimal K value was estimated using the method of Evanno^66^. The number of iterations was set to 500,000 with a 10% of burn-in. Ten independent replicas for each K were performed. Replicas with different patterns were found for K = 4, 8 of the 10 being the same. CLUMPP 1.1.2^67^ was used to summarize the results.

Pairwise F_st_ distances between the different populations were estimated using the SNPs with the Weir and Cockerman (1984) method in the *genet.dist* function of the *hierfstat* R package^68^. Using *boot.ppfst* from the same package, 95% confidence intervals were calculated with 100,000 replications bootstrapping over loci. Any intervals that did not overlap zero were inferred to be significant.

### Estimation of specific mutation rates of ddRAD loci from rodent sequences

A pipeline of several bioinformatic steps was designed to detect orthologues of *Arvicola* ddRAD sequences and estimate their mutation rates, as depicted in Fig. S2. The set of 2874 ddRAD sequences used as seed in this pipeline belonged mostly to a single specimen of *A. scherman* (IBE-C4977; Table S1), except for a few sequences that were missing from this individual and taken from another (IBE-C4969; Table S1). Any other specimen of this or the other species would have produced the same results given the small differences between *Arvicola* sequences compared to the large differences with other rodent species. First, one sequence per locus was used to perform a BLAST search^40^ against the mouse genome using an E-value of 1e^−10^ (80,352 hits). Only loci with single hits and reported E-values lower than 1e^−40^ were considered to ensure as much as possible 1:1 orthology (118 orthologues). Chromosome number and sequence coordinates of each hit were annotated. Mammalian orthologues of each mouse sequence were then downloaded from the EPO suite of the ENSEMBL database (114 alignments). This database includes complete genomes of vertebrate species, with one genome used as reference per species, and multiple genome alignments of these species from which orthologous regions can be obtained^41^. For downloading the orthologous sequences, we entered the chromosome number and coordinates of the mouse sequence fragments found in the script dna_getAncestralSequences.pl from the ENSEMBL Compara API (http://www.ensembl.org/info/docs/api/compara/index.html). Sequences with more than 100 bp and less than 10 unknown nucleotides were kept. In addition, only alignments that contained mouse and rat sequences were considered (86 alignments). From these alignments, sequences from Muroidea species were retained: *Mus pahari*, *Mus caroli*, *Mus spretus*, *Mus musculus*, *Microtus ochrogaster*, *Cricetulus griseus*, *Rattus norvegicus,* and *Peromyscus maniculatus*. The number of species per alignment ranged from 4 to 9; 93% of alignments had at least 6 species and 41% had all 9 Muroidea species. After adding one *A. scherman* sequence to each locus (the same used as seed; Fig. S2), we realigned each set of rodent orthologues using MAFFT 7.130^69^ with the *localpair*, *maxiterate* and *adjustdirectionaccurately* parameters. Gap positions from the final alignments were removed using Gblocks^70^. After this step, invariant alignments were removed (remaining 85 alignments). If any sequence in this set was not orthologous or was too divergent, it could be detected in a phylogenetic tree as an anomalous branch length. We therefore reconstructed a maximum-likelihood phylogenetic tree from each alignment using RAxML version 8.0.19^71^. The trees were visually inspected to ensure that no trees with large branch lengths were present in the final set.

A tree was constructed from the 85 rodent alignments with BEAST version 2.5.2^21^, using the calibrating point of the mouse-rat split at 10.4–14.0 Myr^20^. Unlinked site models (HKY + I) across loci, unlinked clocks (strict clock) and linked trees (Yule) were selected. A strict clock was chosen because the analysis converged better than with a relaxed clock in initial runs, as expected when the mutation rate variation between lineages is low^72^. The calibrated node was modeled using a lognormal prior distribution with minimum and maximum constraints in real space; the offset and the soft maximum were set to 10.4 and 14.0 Myr, respectively, to coincide with the 95th percentile of the probability density distribution with a standard deviation of 1. A total of 75 million generations were run, sampling each 1000 generations. The Tracer v1.7.1 program^73^ was used to check convergence and retrieve the mutation rate of each locus. The TreeAnnotator program from the BEAST package was used to calculate the consensus tree with median heights and 10% burn-in.

### Isolation-with-migration analyses

To estimate divergence times in an IM analysis we used IMa3^43^. Four populations were considered: the Cantabrian and Pyrenean populations of *A. scherman* from the Iberian Peninsula, a sample from the central European distribution of this species, and the *A. amphibius* samples. In this configuration, the population topology was, according to the genomic tree: (((*A. scherman* Cantabrian, *A. scherman* Pyrenean), *A. scherman* central European), *A. amphibius*). After different tests, the final analysis was carried out using 300 randomly selected loci that included the 85 calibrated loci. The mutation rates estimated previously using BEAST2 were included in the corresponding loci of the IMa3 input file after scaling them per alignment, as required by the program. To calculate the population size in demographic units, the generation time was set to 1 year, a value that can be considered adequate for a short-lived species like *Arvicola* sp*.* However, it should be noted that the generation time does not affect the estimation of divergence times^5^. The infinite sites model was used for all loci. Seven loci that did not pass the four gametes test were trimmed, with the longest fragment being selected^74^. The priors in the IMa3 model were adjusted to short loci and recently split populations and species, and they were selected according to their convergence in initial runs: maximum population size mutation rate *4N*µ (q) = 1.5; maximum split time *t*µ = 0.5; and maximum migration rate *m*/µ = 2. Note that all parameters are scaled by the mutation rate µ. Runs were performed with 420 chains (a large number due to the considerable number of loci) on 28 processors (the final run took 265 h). Burn-in was set to ~ 500,000 steps and 15,000 genealogies, sampled every 100 steps, were saved. To ensure proper mixing and convergence, plots of parameter trends and marginal posterior probability distributions of the parameters were checked, and estimates of the first and second halves of the sampled genealogies were compared. Parameter estimates reported were the histogram bins with the highest value and confidence intervals were the 95% highest posterior density intervals (HPD). The IMfig program^45^ was used to prepare the figure with the schematic representation of the generated isolation-with-migration model. Significance of migration rates was based on the log-likelihood-ratio test of the null hypothesis of zero migration^3^.

## Supplementary Information

Supplementary Information.

## Data Availability

ddRAD data and alignments used for calibration are available in Dryad (10.5061/dryad.cz8w9gj5d).
