# Supplementary material for: Divergence time estimation using ddRAD data and an isolation-with-migration model applied to water vole populations of Arvicola
Source: Sci Rep. 2022 Mar 8;12:4065. doi: 10.1038/s41598-022-07877-y (PMC8904462; doi:10.1038/s41598-022-07877-y)
Supplement: Supplementary file 1 — Supplementary Information. [file 41598_2022_7877_MOESM1_ESM.pdf]

Supplementary Information: Tables and Figures

Divergence time estimation using ddRAD data and an isolation-with-migration model applied to water vole populations of *Arvicola*

Alfonso Balmori-de la Puente[1], Jacint Ventura[2,3], Marcos Miñarro[4], Aitor Somoano[4], Jody Hey[5], and Jose Castresana[1*]

1 Institute of Evolutionary Biology (CSIC-Universitat Pompeu Fabra), Passeig Marítim de la Barceloneta 37, 08003 Barcelona, Spain

2 Universitat Autònoma de Barcelona, Facultat de Biociències, Departament de Biologia Animal, de Biologia Vegetal i d'Ecologia, 08193 Cerdanyola del Vallès, Barcelona, Spain

3 Àrea de recerca en petits mamífers, Granollers Museum of Natural Sciences, Palaudàries, 102, 08402 Granollers, Barcelona, Spain

4 Servicio Regional de Investigación y Desarrollo Agroalimentario (SERIDA), Ctra AS-267, PK 19, 33300 Villaviciosa, Asturias, Spain

5 Department of Biology, Center for Computational Genetics and Genomics, Temple University, Philadelphia, PA 19122, USA

* Corresponding author: Jose Castresana

Institute of Evolutionary Biology (CSIC-Universitat Pompeu Fabra), Passeig Marítim de la Barceloneta 37, 08003 Barcelona, Spain

Table S1. Samples used in the study including species name, sample type and geographic information. Samples from the Museum of Southwestern Biology (MSB), the University of Alaska Museum (UAM) and the Museum of Vertebrate Zoology (MVZ) are indicated with their respective codes.

Specimen Code	Species	Sample type	Lat	Lon	Locality	Adm. Division / Country	Population
IBE-C5800	*A. scherman*	Tissue	43.5	-6.7	Coaña	Asturias	*A. s.* Cantabrian
IBE-C5801	*A. scherman*	Tissue	43.5	-6.7	Coaña	Asturias	*A. s.* Cantabrian
IBE-C5796	*A. scherman*	Skull	43.2	-6.6	Santa Marina de Obanca	Asturias	*A. s.* Cantabrian
IBE-C5791	*A. scherman*	Skull	43.2	-6.6	Santa Marina de Obanca	Asturias	*A. s.* Cantabrian
IBE-C5795	*A. scherman*	Skull	43.2	-6.6	Santa Marina de Obanca	Asturias	*A. s.* Cantabrian
IBE-C4973	*A. scherman*	Tissue	43.4	-6.4	La Fajera	Asturias	*A. s.* Cantabrian
IBE-C4971	*A. scherman*	Tissue	43.0	-6.1	Valle de Lago	Asturias	*A. s.* Cantabrian
IBE-C4968	*A. scherman*	Tissue	43.0	-5.9	La Cubilla	León	*A. s.* Cantabrian
IBE-C5802	*A. scherman*	Tissue	43.2	-5.8	Mieres	Asturias	*A. s.* Cantabrian
IBE-C5803	*A. scherman*	Tissue	43.2	-5.8	Mieres	Asturias	*A. s.* Cantabrian
IBE-C4986	*A. scherman*	Tissue	43.4	-5.4	Poreño	Asturias	*A. s.* Cantabrian
IBE-C4979	*A. scherman*	Tissue	43.5	-5.4	Santa Marina	Asturias	*A. s.* Cantabrian
IBE-C6033	*A. scherman*	Skull	42.8	-5.3	La Ercina	León	*A. s.* Cantabrian
IBE-C6027	*A. scherman*	Skull	42.8	-5.3	La Ercina	León	*A. s.* Cantabrian
IBE-C6026	*A. scherman*	Skull	42.8	-5.2	Valporquero de Rueda	León	*A. s.* Cantabrian
IBE-C4965	*A. scherman*	Tissue	43.3	-4.9	Asiego	Asturias	*A. s.* Cantabrian
IBE-C4966	*A. scherman*	Tissue	43.3	-4.9	Asiego	Asturias	*A. s.* Cantabrian
IBE-C2497	*A. scherman*	Skull	43.0	-4.2	Villacantid	Cantabria	*A. s.* Cantabrian
IBE-C2354	*A. scherman*	Skull	43.0	-4.2	Villacantid	Cantabria	*A. s.* Cantabrian
IBE-C4975	*A. scherman*	Tissue	43.3	-2.1	Usurbil	Gipuzkoa	*A. s.* Pyrenean
IBE-C4974	*A. scherman*	Tissue	43.3	-2.1	Usurbil	Gipuzkoa	*A. s.* Pyrenean
IBE-C4969	*A. scherman*	Tissue	43.3	-2.0	Ibaeta	Gipuzkoa	*A. s.* Pyrenean
IBE-C4970	*A. scherman*	Tissue	43.3	-2.0	Ibaeta	Gipuzkoa	*A. s.* Pyrenean
IBE-C4977	*A. scherman*	Tissue	43.3	-1.9	Astigarraga	Gipuzkoa	*A. s.* Pyrenean
IBE-C4976	*A. scherman*	Tissue	43.3	-1.9	Astigarraga	Gipuzkoa	*A. s.* Pyrenean
IBE-C5797	*A. scherman*	Tissue	43.0	-1.3	Burguete	Navarra	*A. s.* Pyrenean
IBE-C5793	*A. scherman*	Skull	43.0	-1.3	Burguete	Navarra	*A. s.* Pyrenean
IBE-C5012	*A. scherman*	Tissue	42.7	0.8	Arrós	Lleida	*A. s.* Pyrenean
IBE-C5010	*A. scherman*	Tissue	42.7	0.8	Arrós	Lleida	*A. s.* Pyrenean
IBE-C5013	*A. scherman*	Tissue	42.7	0.8	Arrós	Lleida	*A. s.* Pyrenean
IBE-C5011	*A. scherman*	Tissue	42.7	0.8	Arrós	Lleida	*A. s.* Pyrenean
IBE-C4356	*A. scherman*	Tissue	42.6	1.2	Ribera de Cardós	Lleida	*A. s.* Pyrenean
MVZ:Mamm:155884	*A. scherman*	Tissue	46.8	8.4	Engelberg	Switzerland	*A. s.* Central Eur.
UAM:Mamm:64164	*A. amphibius*	Tissue	62.2	22.8	Alkkia	Finland	*A. amphibius*
UAM:Mamm:64166	*A. amphibius*	Tissue	62.2	22.8	Alkkia	Finland	*A. amphibius*
MSB:Mamm:288873	*A. amphibius*	Tissue	48.2	89.0	Songinot Gol	Mongolia	*A. amphibius*
MSB:Mamm:289204	*A. amphibius*	Tissue	48.2	89.0	Songinot Gol	Mongolia	*A. amphibius*
MSB:Mamm:148349	*A. amphibius*	Tissue	61.2	132.7	Amga River	Russia	*A. amphibius*
MSB:Mamm:148350	*A. amphibius*	Tissue	61.2	132.7	Amga River	Russia	*A. amphibius*

Table S2. Basic statistics of the bioinformatic analyses of the genomic libraries before and after filtering the exogenous sequences present in every sample.

	PREFILTERED			FILTERED			
Specimen Code	Assembled reads	Assembled loci	Coverage	Assembled reads	Assembled loci	Coverage	Percentage of endogenous DNA
IBE-C5800	3,426,361	87,674	39.1	2,815,345	51,244	54.9	82.2
IBE-C5801	3,799,579	81,871	46.4	3,223,915	51,114	63.1	84.8
IBE-C5796	15,660,996	112,436	139.3	1,342,487	48,108	27.9	8.6
IBE-C5791	8,251,001	245,821	33.6	4,517,558	52,230	86.5	54.8
IBE-C5795	11,122,396	131,273	84.7	7,130,304	51,550	138.3	64.1
IBE-C4973	3,299,873	83,466	39.5	2,698,496	50,987	52.9	81.8
IBE-C4971	3,448,010	87,047	39.6	2,679,942	50,979	52.6	77.7
IBE-C4968	3,290,333	77,114	42.7	2,846,154	50,932	55.9	86.5
IBE-C5802	3,471,619	89,083	39.0	2,832,729	51,462	55.0	81.6
IBE-C5803	4,185,777	87,169	48.0	3,500,468	52,053	67.2	83.6
IBE-C4986	2,295,282	97,802	23.5	1,676,338	51,098	32.8	73.0
IBE-C4979	2,913,053	81,270	35.8	2,411,892	50,148	48.1	82.8
IBE-C6033	3,462,082	101,741	34.0	1,842,996	49,982	36.9	53.2
IBE-C6027	11,799,850	93,577	126.1	1,369,570	44,944	30.5	11.6
IBE-C6026	6,618,081	145,709	45.4	4,690,568	50,731	92.5	70.9
IBE-C4965	2,232,377	80,341	27.8	1,763,455	49,600	35.6	79.0
IBE-C4966	2,976,810	93,960	31.7	2,120,171	49,994	42.4	71.2
IBE-C2497	4,341,737	139,354	31.2	3,063,097	53,665	57.1	70.6
IBE-C2354	6,420,061	126,652	50.7	4,776,479	53,273	89.7	74.4
IBE-C4975	3,236,023	79,867	40.5	2,596,083	50,195	51.7	80.2
IBE-C4974	1,948,901	86,981	22.4	1,366,608	47,991	28.5	70.1
IBE-C4969	2,693,192	82,608	32.6	2,229,766	51,417	43.4	82.8
IBE-C4970	3,019,630	86,390	35.0	2,426,066	51,681	46.9	80.3
IBE-C4977	2,076,792	91,064	22.8	1,520,958	50,286	30.2	73.2
IBE-C4976	1,760,633	74,562	23.6	1,455,469	49,704	29.3	82.7
IBE-C5797	3,785,135	92,407	41.0	2,942,600	51,069	57.6	77.7
IBE-C5793	10,306,646	176,053	58.5	7,212,609	56,014	128.8	70.0
IBE-C5012	2,891,562	96,721	29.9	2,208,599	51,682	42.7	76.4
IBE-C5010	2,029,993	82,628	24.6	1,539,931	48,875	31.5	75.9
IBE-C5013	6,520,367	168,006	38.8	4,754,722	52,057	91.3	72.9
IBE-C5011	7,500,393	124,048	60.5	5,757,512	53,542	107.5	76.8
IBE-C4356	1,985,169	76,003	26.1	1,566,069	48,586	32.2	78.9
MVZ:Mamm:155884	8,010,044	106,245	75.4	6,434,560	53,952	119.3	80.3
UAM:Mamm:64164	6,352,507	137,650	46.1	3,964,195	43,649	90.8	62.4
UAM:Mamm:64166	1,167,434	54,473	21.4	659,945	29,914	22.1	56.5
MSB:Mamm:288873	4,716,459	79,076	59.6	3,435,332	44,860	76.6	72.8
MSB:Mamm:289204	5,779,469	72,191	80.1	3,982,328	42,853	92.9	68.9
MSB:Mamm:148349	8,358,565	98,527	84.8	6,117,091	46,011	132.9	73.2
MSB:Mamm:148350	5,756,804	84,216	68.4	4,365,322	45,416	96.1	75.8
Average	4,946,436	102,387	47.4	3,175,326	49,586	63.4	71.3
Sum	192,910,996	3,993,076		123,837,729	1,933,848		

Table S3. Pairwise F_{st} values and 95% confidence intervals between the different populations analyzed.

	A. scherman Pyrenean	*A. scherman* Cantabrian	*A. scherman* Central European
A. scherman Cantabrian	0.27 (0.26 - 0.29)		
A. scherman European	0.30 (0.26 - 0.33)	0.34 (0.30 - 0.37)	
A. amphibius	0.65 (0.63 - 0.67)	0.65 (0.63 - 0.67)	0.66 (0.63 - 0.68)

Table S4. Mutation rates estimated in the BEAST2 analysis using orthologues from other species of rodents and a fossil calibration point.

Locus name	Rate (mutations/site/yr)	Locus name	Rate (mutations/site/yr)
*Arvicola*_45428	2.98E-10	*Arvicola*_50749	2.46E-09
*Arvicola*_42847	5.11E-10	*Arvicola*_2944	2.47E-09
*Arvicola*_27128	7.91E-10	*Arvicola*_18146	2.49E-09
*Arvicola*_40724	8.69E-10	*Arvicola*_50803	2.52E-09
*Arvicola*_3538	9.46E-10	*Arvicola*_11593	2.58E-09
*Arvicola*_13117	9.84E-10	*Arvicola*_5283	2.60E-09
*Arvicola*_21281	1.06E-09	*Arvicola*_22238	2.63E-09
*Arvicola*_27599	1.16E-09	*Arvicola*_45768	2.71E-09
*Arvicola*_6155	1.23E-09	*Arvicola*_28997	2.72E-09
*Arvicola*_11376	1.25E-09	*Arvicola*_9776	2.79E-09
*Arvicola*_1295	1.29E-09	*Arvicola*_25695	2.80E-09
*Arvicola*_25554	1.34E-09	*Arvicola*_23832	2.93E-09
*Arvicola*_22032	1.51E-09	*Arvicola*_15494	2.95E-09
*Arvicola*_15252	1.52E-09	*Arvicola*_14543	2.95E-09
*Arvicola*_45150	1.57E-09	*Arvicola*_17227	3.02E-09
*Arvicola*_34719	1.57E-09	*Arvicola*_46856	3.04E-09
*Arvicola*_8255	1.60E-09	*Arvicola*_23384	3.14E-09
*Arvicola*_25127	1.64E-09	*Arvicola*_7123	3.18E-09
*Arvicola*_5164	1.67E-09	*Arvicola*_40876	3.25E-09
*Arvicola*_45270	1.75E-09	*Arvicola*_17389	3.29E-09
*Arvicola*_2503	1.82E-09	*Arvicola*_7454	3.36E-09
*Arvicola*_36177	1.89E-09	*Arvicola*_26217	3.44E-09
*Arvicola*_38973	1.91E-09	*Arvicola*_34108	3.45E-09
*Arvicola*_539	1.91E-09	*Arvicola*_28823	3.50E-09
*Arvicola*_29000	1.94E-09	*Arvicola*_28389	3.52E-09
*Arvicola*_323	1.96E-09	*Arvicola*_23533	3.53E-09
*Arvicola*_35426	2.01E-09	*Arvicola*_6124	3.71E-09
*Arvicola*_14647	2.13E-09	*Arvicola*_37324	3.78E-09
*Arvicola*_27258	2.14E-09	*Arvicola*_14628	3.79E-09
*Arvicola*_27347	2.15E-09	*Arvicola*_19415	3.83E-09
*Arvicola*_43484	2.16E-09	*Arvicola*_45177	3.94E-09
*Arvicola*_16676	2.17E-09	*Arvicola*_5737	4.11E-09
*Arvicola*_45394	2.20E-09	*Arvicola*_49442	4.17E-09
*Arvicola*_13652	2.23E-09	*Arvicola*_14128	4.18E-09
*Arvicola*_45826	2.28E-09	*Arvicola*_34910	4.19E-09
*Arvicola*_8901	2.35E-09	*Arvicola*_51131	4.22E-09
*Arvicola*_11509	2.36E-09	*Arvicola*_6366	4.23E-09
*Arvicola*_49832	2.36E-09	*Arvicola*_30646	4.29E-09
*Arvicola*_32730	2.38E-09	*Arvicola*_30182	4.45E-09
*Arvicola*_40032	2.39E-09	*Arvicola*_11439	4.58E-09
*Arvicola*_8735	2.39E-09	*Arvicola*_5712	5.02E-09
*Arvicola*_46446	2.43E-09	*Arvicola*_50070	5.33E-09
*Arvicola*_21065	2.45E-09		

Table S5. Marginal peak estimates of population size scaled by the mutation rate ($4N\mu$) and population migration rate (2Nm) for the first half (Set0), the second half (Set1) and all the sampled genealogies (All). q0, q1, q2 and q3 represent the population size mutation rate of the Cantabrian, Pyrenean, and central European populations of *A. scherman*, and the *A. amphibius* populations, respectively, whereas q4 corresponds to the ancestral population size mutation rate of the first two, q5 of the first three, and q6 of the four.

Parameter	Set0	Set1	All
q0	0.058	0.057	0.058
q1	0.058	0.055	0.057
q2	0.217	0.202	0.212
q3	0.066	0.067	0.066
q4	0.559	0.604	0.558
q5	0.125	0.135	0.133
q6	0.221	0.233	0.221
2N0M0>1	0.041	0.035	0.038
2N0M0>2	0.041	0.039	0.040
2N0M0>3	0.002	0.001	0.002
2N1M1>0	0.041	0.039	0.040
2N1M1>2	0.036	0.038	0.038
2N1M1>3	0.004	0.006	0.006
2N2M2>0	0.082	0.104	0.099
2N2M2>1	0.110	0.000	0.000
2N2M2>3	0.033	0.036	0.035
2N3M3>0	0.000	0.000	0.000
2N3M3>1	0.000	0.000	0.000
2N3M3>2	0.027	0.045	0.036
2N2M2>4	0.120	0.000	0.103
2N3M3>4	0.055	0.055	0.055
2N4M4>2	0.011	0.291	0.307
2N4M4>3	0.149	0.146	0.147
2N3M3>5	0.055	0.056	0.056
2N5M5>3	0.042	0.010	0.005

Table S6. Population sizes and 95% highest posterior density intervals of *Arvicola* sp. populations. q0, q1, q2 and q3 represent the population size of the Cantabrian, Pyrenean, and central European populations of *A. scherman*, and the *A. amphibius* populations, respectively, whereas q4 corresponds to the ancestral population size of the first two, q5 of the first three, and q6 of the four.

Population	Population size and 95% confidence interval
q0	50,859 (29,723 - 74,637)
q1	50,859 (28,402 - 74,637)
q2	186,923 (93,131 - 437,915)
q3	58,785 (38,970 - 79,921)
q4	492,077 (247,690 - 1,064,074)
q5	115,588 (66,711 - 190,886)
q6	194,849 (94,452 - 280,715)

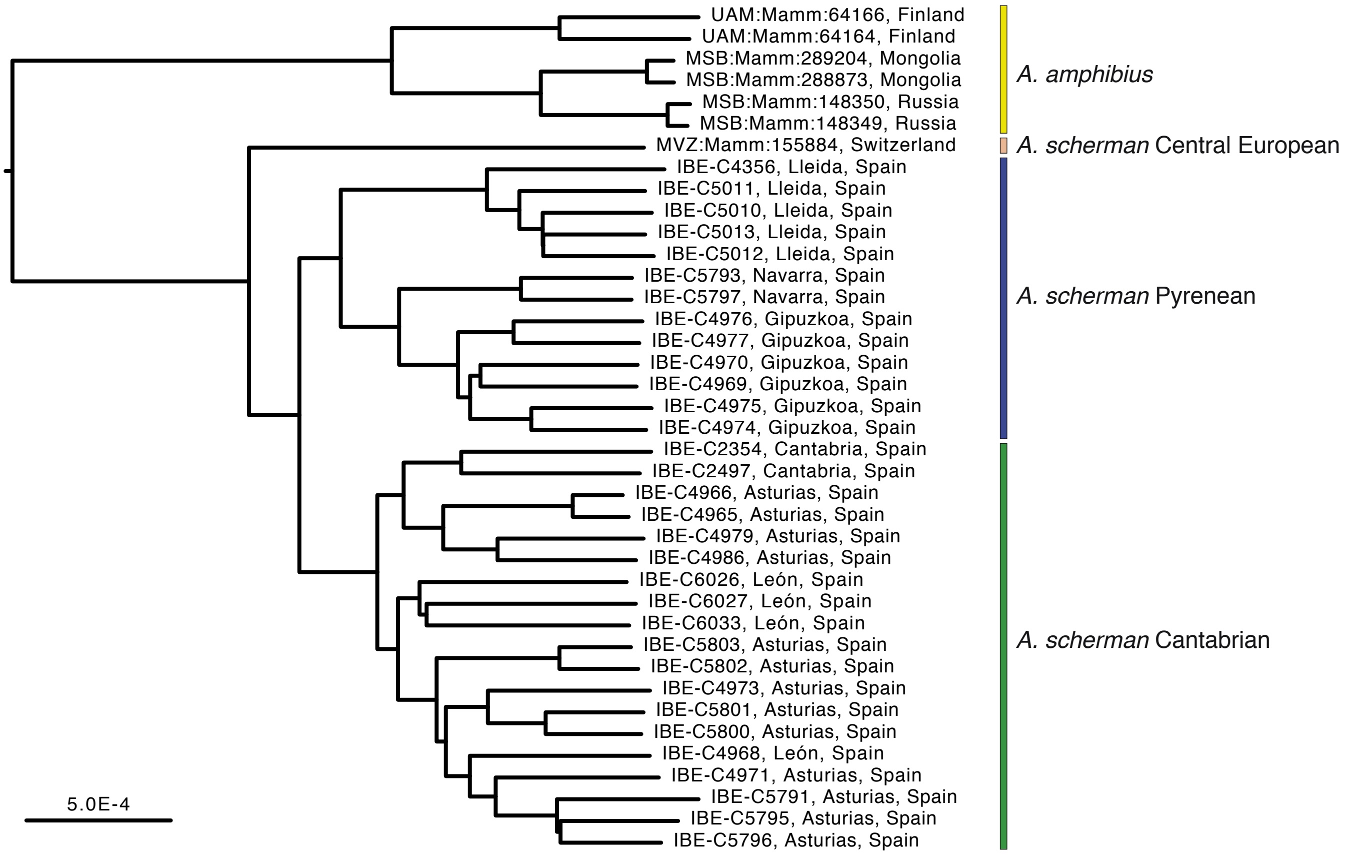

Figure S1. Genomic tree of the *Arvicola* samples obtained from 3,361 loci. The scale is in substitutions per position and mid-point rooting was used to represent the tree.

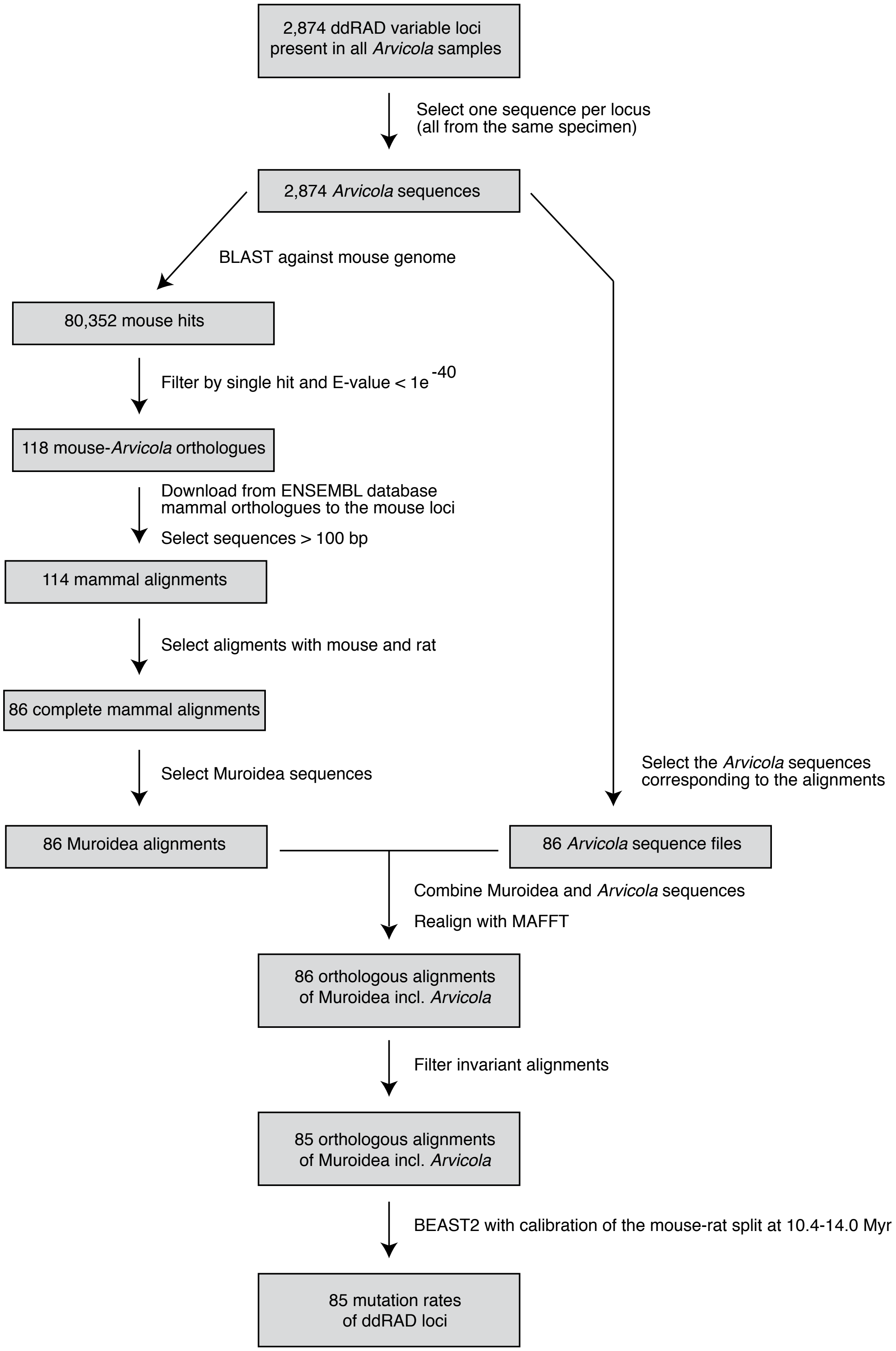

Figure S2. Pipeline used to estimate mutation rates of ddRAD loci.

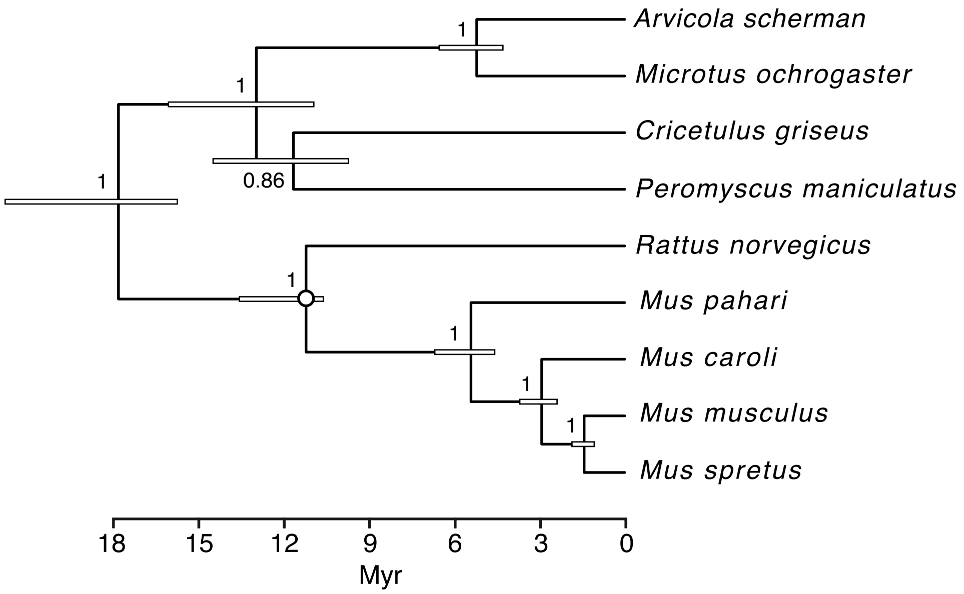

Figure S3. Calibrated tree of Muroidea reconstructed using BEAST2 and 85 ddRAD loci. The white circle shows the mouse-rat calibration prior (10.4–14.0 Myr). The node bars indicate the 95% confidence interval of the corresponding divergence times and the posterior probabilities are specified for each clade.

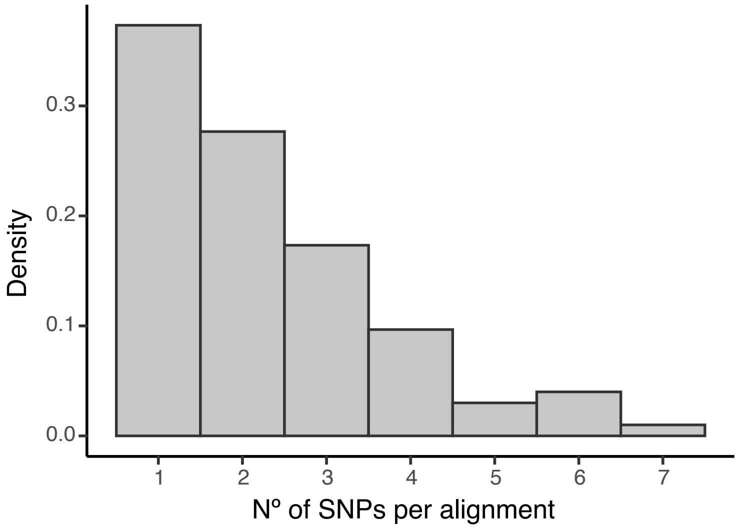

Figure S4. Distribution of polymorphic positions per locus of the loci used in the IMa3 model.

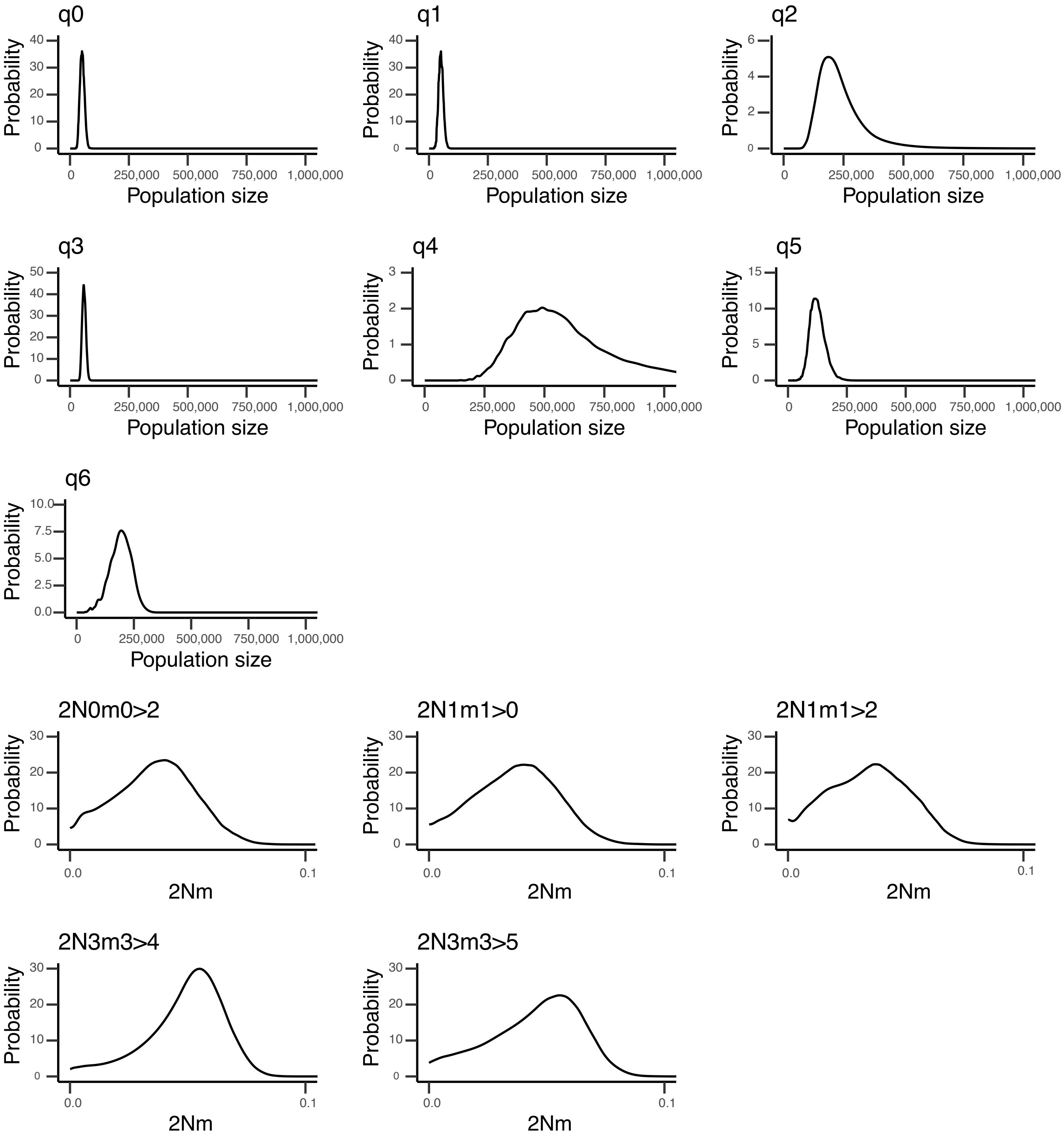

Figure S5. Marginal posterior probability histograms of the isolation-with-migration model. Top rows: population size. Bottom rows: population migration rate. For migration rates only significant values are shown. q0, q1, q2 and q3 represent the population size of the Cantabrian, Pyrenean, and central European populations of *A. scherman*, and the *A. amphibius* populations, whereas q4 corresponds to the ancestral population size of the first two, q5 of the first three, and q6 of the four.

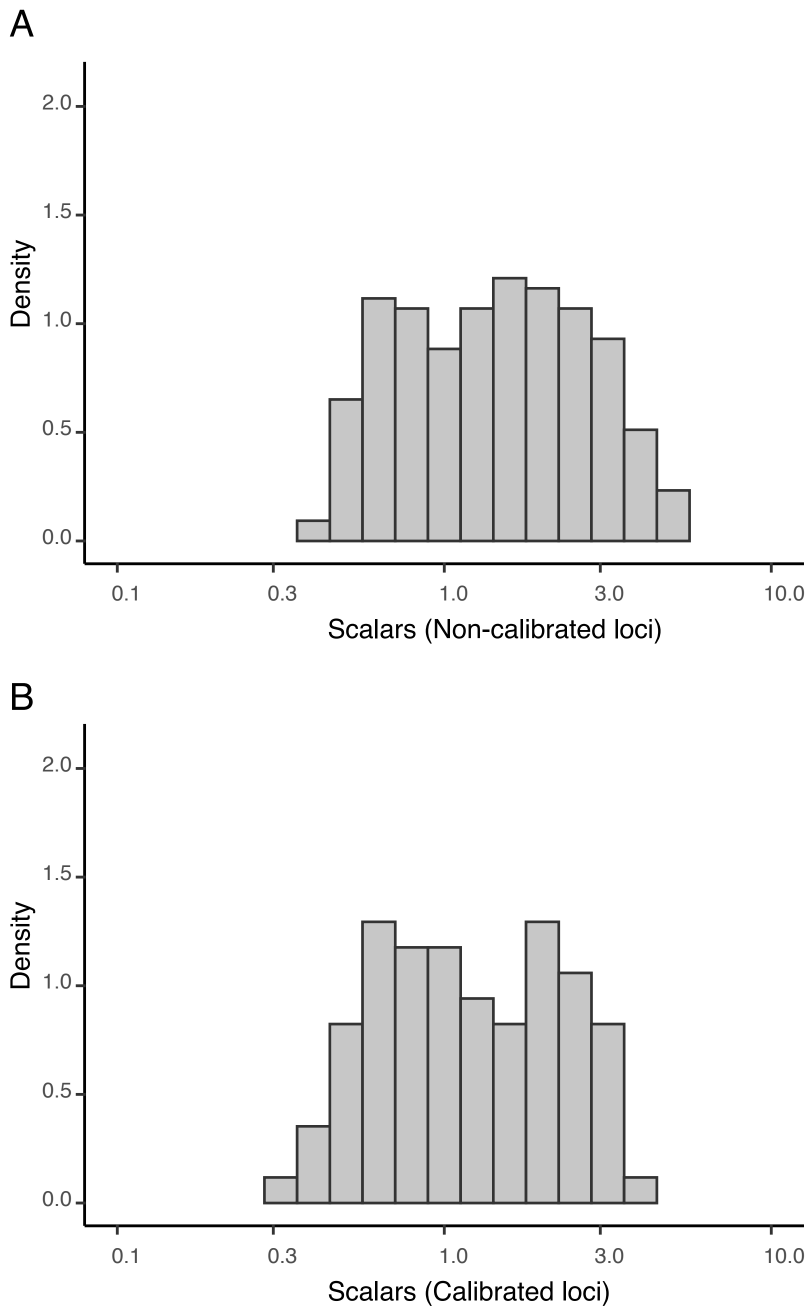

Figure S6. Histograms of mutation rate scalars ($\log_{10}$) estimated by IMa3 of (A) non-calibrated and (B) calibrated loci.
